# The effect of a cyclic uniaxial strain on urinary bladder cells

**DOI:** 10.1007/s00345-017-2013-9

**Published:** 2017-02-23

**Authors:** Dorien Tiemessen, Paul de Jonge, Willeke Daamen, Wout Feitz, Paul Geutjes, Egbert Oosterwijk

**Affiliations:** 1 Department of Urology, Radboud Institute for Molecular Life Sciences, Radboud University Medical Center, route267, Geert Grooteplein 16, 6525 GA Nijmegen, The Netherlands; 2 Department of Biochemistry, Radboud Institute for Molecular Life Sciences, Radboud University Medical Center, Nijmegen, The Netherlands

**Keywords:** Urinary bladder, Augmentation, Bioreactor, Tissue engineering, Mechanical stimulation, Scaffold

## Abstract

**Purpose:**

Pre-conditioning of a cell seeded construct may improve the functional outcome of a tissue engineered construct for augmentation cystoplasty. The precise effects of mechanical stimulation on urinary bladder cells in vitro are not clear. In this study we investigate the effect of a cyclic uniaxial strain culture on urinary bladder cells which were seeded on a type I collagen scaffold.

**Methods:**

Isolated porcine smooth muscle cells or urothelial cells were seeded on a type I collagen scaffolds and cultured under static and dynamic conditions. A uniform cyclic uniaxial strain was applied to the seeded scaffold using a Bose Electroforce Bio-Dynamic bioreactor. Cell proliferation rate and phenotype were investigated, including SEM analysis, RT-PCR and immunohistochemistry for α-Smooth muscle actin, calponin-1, desmin and RCK103 expression to determine the effects of mechanical stimulation on both cell types.

**Results:**

Dynamic stimulation of smooth muscle cell seeded constructs resulted in cell alignment and enhanced proliferation rate. Additionally, expression of α-Smooth muscle actin and calponin-1 was increased suggesting differentiation of smooth muscle cells to a more mature phenotype.

**Conclusions:**

Mechanical stimuli did not enhance the proliferation and differentiation of urothelial cells. Mechanical stimulation, i.e., preconditioning may improve the functional in vivo outcome of smooth muscle cell seeded constructs for flexible organs such as the bladder.

**Electronic supplementary material:**

The online version of this article (doi:10.1007/s00345-017-2013-9) contains supplementary material, which is available to authorized users.

## Introduction

For patients who need bladder reconstruction, a tissue engineered bladder may be an alternative to current procedures in which autologous bowel tissue is used. The first clinical studies with cell seeded bladder-sized constructs illustrated that scaffolds implanted in patients who had a normal bladder cycle regenerated properly, while patients with abnormal cycles responded poorly [1]. Bladder regeneration studies in animals indicated slow smooth muscle cell ingrowth in scaffolds, probably due to the limited migration from adjacent tissue [2]. This suggests that adequate conditioning of the tissue engineered construct may be needed to improve the functional outcome of the regenerated tissue for flexible organs.

It has become clear that mechanical stimulation is equally important in cellular behavior as genetic and chemical signals [3]. By providing mechanotransduction, cell proliferation and differentiation can be influenced and it may lead to extracellular matrix (ECM) production [4–6]. Therefore, it is important to investigate the behavior and phenotype of cells in constructs while under defined mechanical strain before implantation.

A bioreactor can apply mechanical stimuli under controlled in vitro conditions [7, 8]. Initially vacuum suction (Seliktar et al., 2000) and mechanical stretch was used [5]. Thereafter different bioreactor systems using hydrostatic pressures have been developed and used to study urinary bladder tissue engineering [9]. Hydrostatic pressure on human bladder smooth muscle cells on aligned nanofibrous scaffolds resulted in functional improvement of the engineered tissue [10]. Although this setting simulated in vivo conditions, the exact impact of the mechanical stimulation on the urinary bladder cells is not clear. Moreover, whether mechanical stimulation of cells seeded on other materials such as collagen also leads to functional improvement is currently unclear. In this study we investigated the effect of a long-term controlled cyclic uniaxial strain on urinary bladder cells which were seeded on a type I collagen scaffold mimicking the filling and emptying of the bladder to assess whether this pre-conditioning step is beneficial in urinary bladder tissue engineering.

## Materials and methods

Products were purchased from Life technologies (Carlsbad; USA) unless otherwise indicated.

### Scaffolds preparation and characterisation

For the construction of collagen scaffolds, insoluble type I collagen fibrils were purified from pulverized bovine Achilles tendon, as previously described [11]. In short, a 0.5% (w/v) type I collagen suspension was made by swelling and subsequent homogenization in 0.25 M acetic acid at 4 °C. The collagen suspension was deaerated, poured into six-well plates, frozen at −20 °C and lyophilized. The dried collagen matrices were stabilized using 1-ethyl-3-(3-dimethyl aminopropyl) carbodiimide (Fluka, Sigma–Aldrich; St. Louis, USA) and *N*-hydroxysuccinimide (Fluka, Sigma–Aldrich; St. Louis, USA) crosslinking in 50 mM 2-morpholinoethane sulphonic acid (MES, pH 5.0) containing 40% (v/v) ethanol for 4 h at 21 °C. After cross-linking, the scaffolds were washed consecutively in 0.1 M Na_2_HPO_4_, 1 M NaCl, 2 M NaCl, demineralized water, disinfected by 70% ethanol washings and stored at −20 °C. The degree of crosslinking of the scaffolds was determined by 2,4,6-trinitrobenzene sulfonic acid (TNBS) analysis in triplicate [12]. Collagen strips were cut to match the bioreactor dimensions (length 2.5 cm, width 1 cm), and washed in 70% ethanol [at least 2 times 1 h and 1 time overnight (o/n)], followed by washings in sterile phosphate-buffered saline (PBS; pH 7.4, at least 2 times 1 h and 1 time o/n), and an o/n incubation in medium.

### Cell isolation

Porcine bladders were collected from a local abattoir. After opening the bladder, ~1 cm^2^ pieces were collected under aseptic conditions and tissue specimens were transferred to transport medium HBSS^+Mg+Ca^, 10 mM HEPES; pH 7.6, aprotinin 1 μg/ml (Roche; Basel, Switzerland), 100 U/ml penicillin and 100 μg/ml streptomycin (P/S). Tissue specimens were incubated 16 h at 4 °C in stripper medium HBSS^−Mg−Ca^, 10 mM HEPES; pH 7.6, aprotinin 1 µg/ml (Roche; Basel, Switzerland), P/S and 2.4 U/ml dispase II (Sigma–aldrich; St Louis, USA). Urothelial cells (UC) were isolated by scraping the urothelial sheet using tweezers. Urothelial sheets were collected in a 15 ml tube and incubated for 20 min at 37 °C with 100 U/ml collagenase type IV (Sigma–Aldrich; St Louis, USA) prepared in transport medium. Cells were collected by centrifugation at 400*g* for 8 min. and seeded in Primaria flasks (BD Falcon^®^, US; 1 T75 per cm^2^ tissue specimen). Cells were cultured in keratinocyte serum-free medium (KSFM) supplemented with 50 μg/ml bovine pituitary extract, 5 ng/ml epidermal growth factor, 30 ng/ml cholera toxin (Sigma–Aldrich; St. Louis, USA) and P/S (UC medium).

For the isolation of SMC, the remaining tissue was cut into small pieces (~2 mm^2^) and incubated for 1.5 h at 37 °C with 1.5 U/ml liberase enzyme (Roche; Basel, Switzerland) diluted in HBSS^+Ca+Mg^ and P/S. After vigorous resuspension, the material was pushed through a 70 μm cell strainer (BD Falcon®, USA) to remove undigested particles. Cells were collected by centrifugation and cultured in smooth muscle cell medium (SMCM, Sciencell; Carlsbad, USA), supplemented with 2% (v/v) fetal bovine serum, 1% (v/v) smooth muscle growth supplement and P/S (2 T75 per cm^2^ tissue specimen). Cultures were maintained at 37 °C in a humidified atmosphere of 5% (v/v) CO_2_ in air. Medium was changed three times a week and cells were split when 100% confluence was reached. Cells harvested from one porcine bladder were used to prevent the influence of individual differences between animals.

Cells from passage 1–3 were used.

### Bioreactor culture

**Fig. 1:**
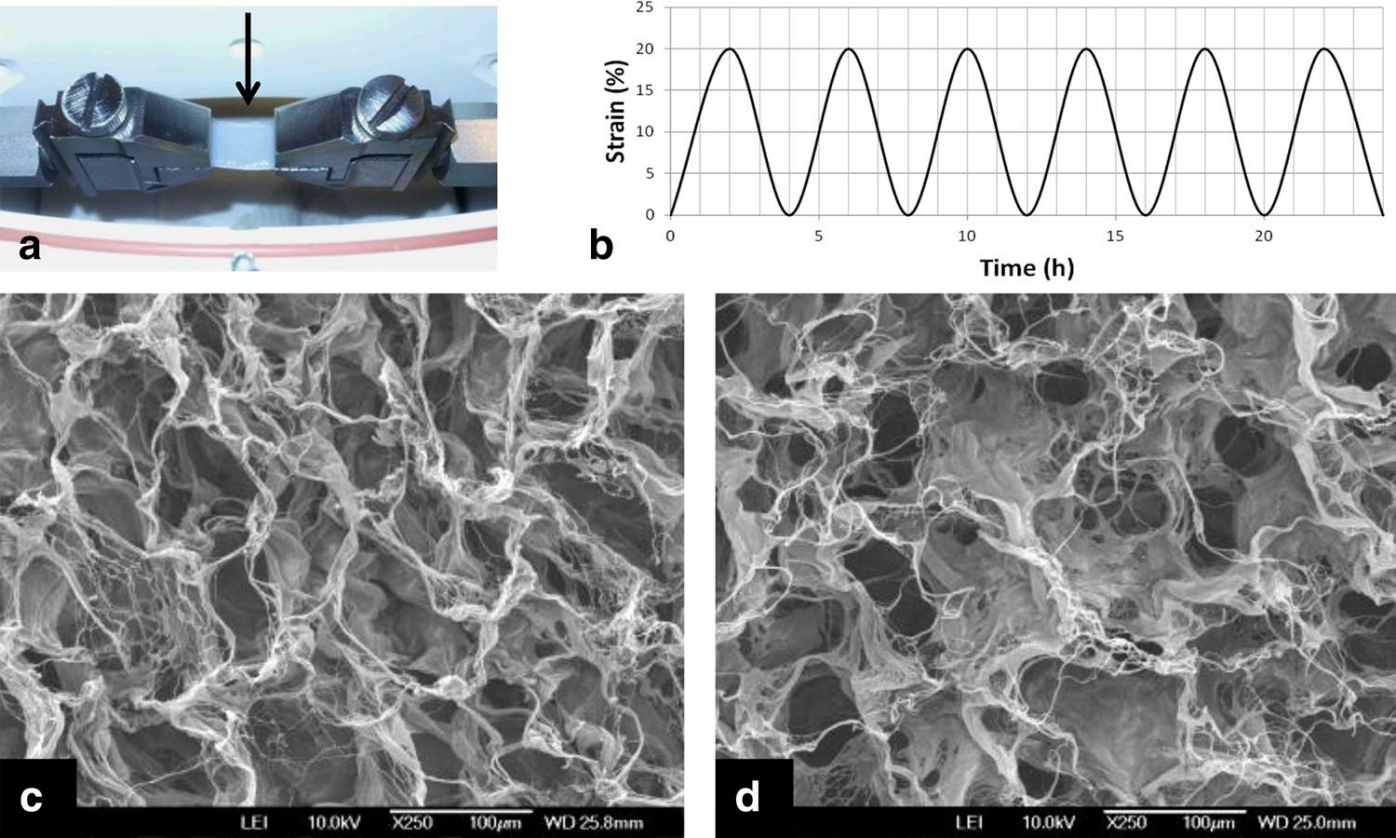
Overview of the experimental setting with a strip of scaffold clamped in the Bose Electroforce Bio-Dynamic bioreactor (*arrow*) (**a**). Overview bioreactor program with a continuous 0.3 µm/s cycle strain (20% full stretch and fold within 4 h) (**b**). Scanning electron microscopic pictures of the used type I collagen scaffold (×250); **c, d** the air and pan side, respectively, showing a typical honeycomb structure

Collagen scaffold strips were placed in a 6-well plate and seeded statically with 1 to 1.5 × 10^6^ SMC or UC in a volume of 100 μl of SMC medium or UC medium. After 1 h the volume was increased to 2.5 ml SMC medium or UC medium. One day after seeding, scaffolds were placed in a Bose Electroforce Bio-Dynamic bioreactor (Fig. 1a). Subsequently the bioreactor chamber was filled with 200 ml RPMI supplemented with 10% FCS, 2 mM glutamin, 100 U/ml penicillin and 100 µg/ml streptomycin and cultured under dynamic conditions. A cyclic uniaxial strain was applied with a continuous 0.3 µm/s cycle strain (20% full stretch followed by folding in 4 h) (Fig. 1b). Control scaffolds were cultured under static conditions in a T75 flask. After 6 days of culture, scaffolds were harvested and processed for evaluation.

### Scanning electron microscopy

Scanning electron microscopy (SEM) was performed on isolated cells (in triplicate) and cells cultured on scaffolds (once for every static/dynamic sample). Materials were washed three times for 1 h with PBS and fixed for at least 1 h in 2% (v/v) glutaraldehyde (Merck, Kenilworth, New Jersey, USA) in 0.1 M phosphate buffer (PB), pH 7.4 at 21 °C. Scaffolds were washed three times (1 h) using 0.1 M PB. For dehydration scaffolds were washed for at least 1 h in increasing concentrations of ethanol [30, 50, 70, 100% (v/v)] and one additional o/n washing step in 100% ethanol. Scaffolds were critical point dried (CPD) using liquid CO_2_. After drying, gold sputtering was performed prior to SEM analysis (JEOL JSM-6310; Tokyo, Japan).

### Reverse transcriptase polymerase chain reaction (RT-PCR)

Biopsies from scaffolds (one for every static/dynamic sample) with cultured cells were minced, immersed in 0.5 ml Trizol and stored (−80 °C). RNA was isolated by chloroform and isopropanol precipitation and treated with DNase I. RT reaction was performed in Perkin Elmer thermal cycler using Super Script II Reverse Transcriptase according to the manufacturer’s description (Life Technologies; Carlsbad, USA). In brief, cDNAs were amplified by semi-quantitative real-time PCR using SYBR-Green PCR Master Mix (Roche; Basel, Switzerland) using a Light Cycler®480 Real Time PCR System. Finally, gene expression levels of alpha smooth muscle actin (ACTA2), calponin (CNN1), desmin (Desm) and type III collagen (Col3A1) were examined and HPRT (housekeeping gene) was used as a control (see Table 1 for primer sequences).

**Table 1:** Primer sequences used for polymerase chain reaction (PCR) amplification

Gene name	Primer sequence (5′–3′)	
ACTA2	F: GCA TGC AGA AGG AGA TCA CA	R: TTG CTG GAA GGT GGA CAG A
CNNl	F: ACT TCA TCA AGG CCA TCA CC	R: TCA CCT TGT TCC CTT TCG TC
Desm	F: TAG CCT CTG GCT TTG GAG AA	R: TGC TGA AGA CTC TGC CCT TT
Col3Al	F: GGA GGA GGA ATC GGA GGC TA	R: CTT TTC CGG CAG GAC CAG AT
HPRT	F: CTC AAC TTT AAC TGG AAA GAA TGT C	R: TCC TTT TCA CCA GCA AGC T

*F* forward primer, *R* reverse primer, *ACTA2* α-SMA, *CNNl* calponin, *Desm* desmin, *Col3Al* collagen3al, *HPRT* housekeeping gene

^a^PCR conditions were denaturation at 95 °C for 5 min; 45 PCR cycles (denaturing: 95 °C for l0 s; annealing: 60 °C for 20 s; extension: 72 °C for 20 s)

### Immunohistochemistry

For histological evaluation, the scaffolds were embedded in Tissue-Tek (O.C.T. Compound) and snap frozen in dry-ice cooled isopentane. Cryostat section (5 μm) were cut and fixed for 10 min in 100% acetone (−20 °C) followed by a blocking step of 30 min with 10% (v/v) goat serum in 1% (w/v) bovine serum albumin/phosphate buffered saline (BSA/PBS). Sections were incubated with one of the following mouse anti-human antibodies: alpha smooth muscle actin (α-SMA, Sigma-Adrich; St. Louis, USA; 1:8000), desmin (BioGenex; Fremont, USA; 1:200), calponin-1 (CNN1, Abcam; Cambridge, UK; 1:100), RCK103 (Cytokeratin 5 and others, Nordic MUbio; Susteren, The Netherlands; 1:1) and rabbit anti-bovine type I collagen (EMD Millipore, Germany; 1:100), all diluted in 1% (w/v) BSA/PBS, for 1 h. After washing (PBS, 3 times), sections were incubated with goat-anti-mouse-Alexa 594 (1:200) and goat-anti-rabbit-Alexa 488 (1:200) for 1 h. After rinsing with PBS (3 times), slides were incubated with 4′,6-diamidino-2-phenylindole (DAPI; 1:200) for 20 min and 21 °C. Finally, slides were mounted with fluorescent mounting medium (Dako; Glostrup, Denmark) and evaluated. Porcine bladder tissue was used as control tissue. Primary antibody was omitted as negative control. All anti-human primary antibodies had porcine cross-reactivity or were tested for cross-reactivity on porcine bladder tissue.

### WST-1 proliferation assay

After culture, a transverse part of every scaffold (10 × 3 mm) was incubated in 500 μl medium with 50 μl cell proliferation reagent WST-1 (Roche; Basel, Switzerland). After 2 h the absorbance at 450 nm was determined.

## Results

### Characterization scaffold

Scanning electron microscopy (SEM) analysis of the type I collagen scaffold showed a highly porous and interconnective network with pores ranging between 50 and 100 μm for both the air and pan side (Fig. 1c, d). The degree of crosslinking of the scaffolds was 72 ± 12% by TNBS analyses.

### Characterization of bladder-derived smooth muscle and urothelial cells

Primary urothelial cell cultures formed cobblestoned epitheloid monolayers. Phenotypic analysis revealed a homogeneous RCK103-positive cell population (Cytokeratin 5 and others) (Online Resource 1a and b). Isolated SMC showed a typical spindle cell shape and expressed α-SMA (Online Resource 1c and d), smoothelin, and desmin over a number of passages. UC contamination in the SMC culture was negligible as judged by RCK103 staining. There were no SMC present in the used UC culture.

### Evaluation of bioreactor cultured scaffolds

**Fig. 2:**
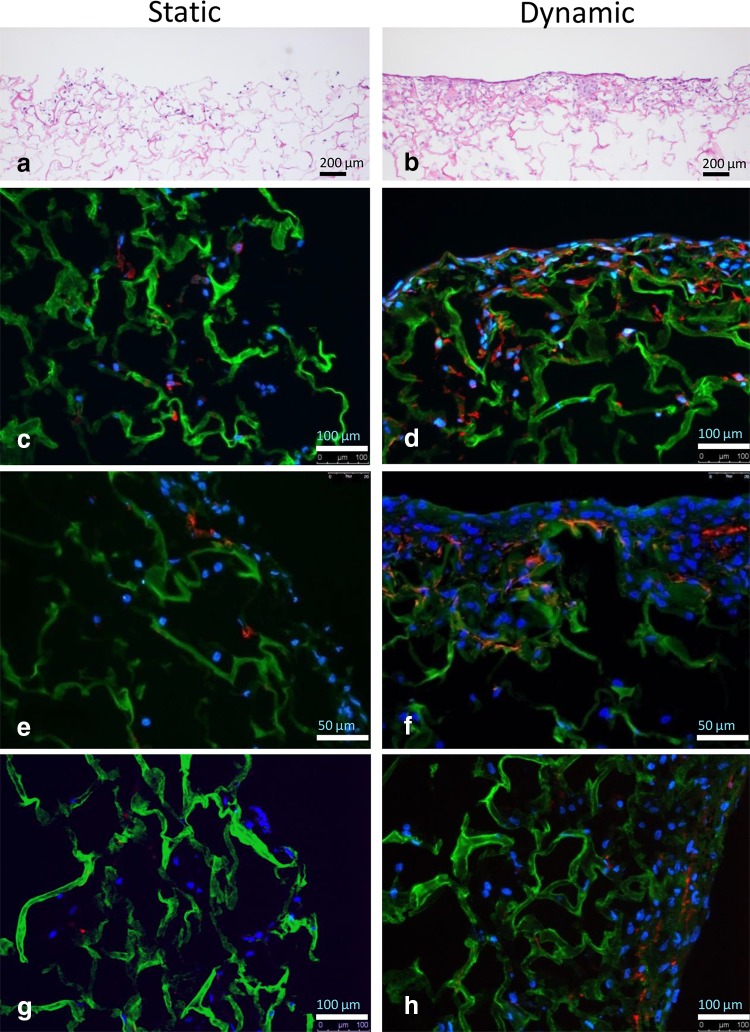
Representative Hematoxylin & Eosin (H&E) staining and immunofluorescence staining of the smooth muscle cell (SMC) seeded constructs cultured under static (**a, c, e**, and **g**) and dynamic (**b, d, f**, and **h**) conditions. **a, b** H&E staining, **c, d** α-SMA, **e, f** calponin-1 and **g, h** desmin staining. *Green* collagen, *blue* nuclear DAPI stain, *red* cell surface marker

**Fig. 3:**
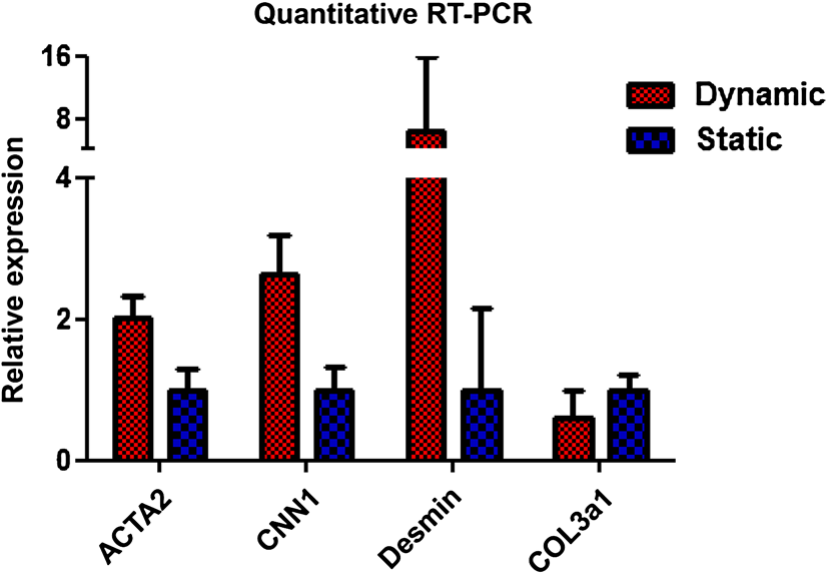
Quantitative RT-PCR data for α-SMA (ACTA2), calponin1 (CNN1), desmin (Desm) and collagen (Col3A1) of smooth muscle cell (SMC) seeded scaffold which were cultured under static and dynamic conditions. The relative expression of the different scaffolds was corrected for the internal HPRT control and the static conditions were set to 1. *Bars* represent the mean ± SD for 3 (SMC) separate experiments. None of the tested markers was expressed by the UC

**Fig. 4:**
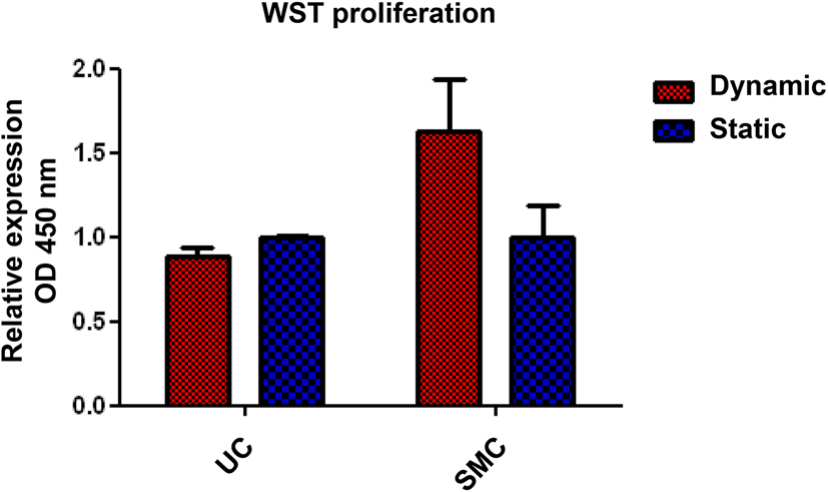
Proliferation (WST-1 cell proliferation assay) of urothelial cell (UC) and smooth muscle cell (SMC) seeded scaffolds which were cultured under static and dynamic conditions. The relative expression of the static conditions was set to 1. *Bars* represent the mean ± SD for 2 (UC) or 3 (SMC) separate experiments

**Fig. 5:**
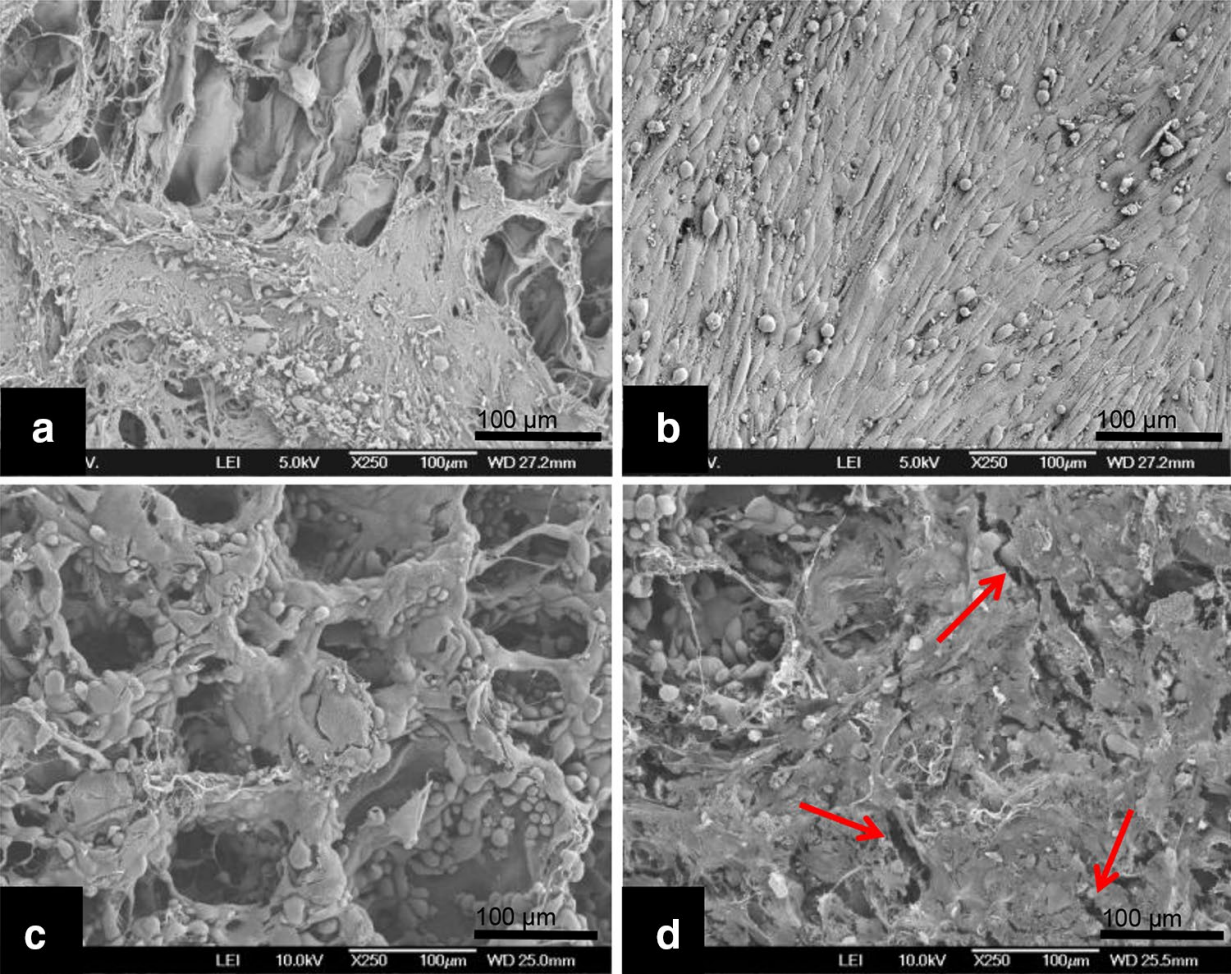
Representative scanning electron microscopy (SEM) analysis of the smooth muscle cell (SMC) and urothelial cell (UC) seeded constructs. SEM of SMC cultured under static (**a**) and dynamic (**b**) conditions. SEM of UC cultured under static (**c**) and mechanical condition (**d**). Please note aligned SMC in (**b**). *Red arrows* point to small ruptures (**d**)

Microscopic evaluation (H&E staining) of the constructs revealed a much denser SMC layer on the scaffold surface when uniaxial strain was applied (Fig. 2a, b) compared to static culture conditions. Phenotypic analysis showed α-SMA expression, regardless of culture conditions. However, relatively more α-SMA positive cells were present in the SMC seeded scaffold cultured under dynamic conditions compared to static culture conditions (Fig. 2c, d). The immunohistochemical analyses demonstrated more intense desmin and calponin1 stainings in the dynamic cultured SMC (Fig. 2e–h). Although a limited number of data is available, the RT-PCR data showed similar results, with a trend of higher expression of ACTA2, CNN1 and desmin levels in dynamic cultures (Fig. 3). WST-1 cell proliferation assays of the SMC seeded scaffolds cultured under dynamic conditions showed increased cell proliferation compared to static cultures (Fig. 4). SEM analysis revealed SMC alignment when the seeded construct was exposed to mechanical stimulation (Fig. 5a, b).

**Fig. 6:**
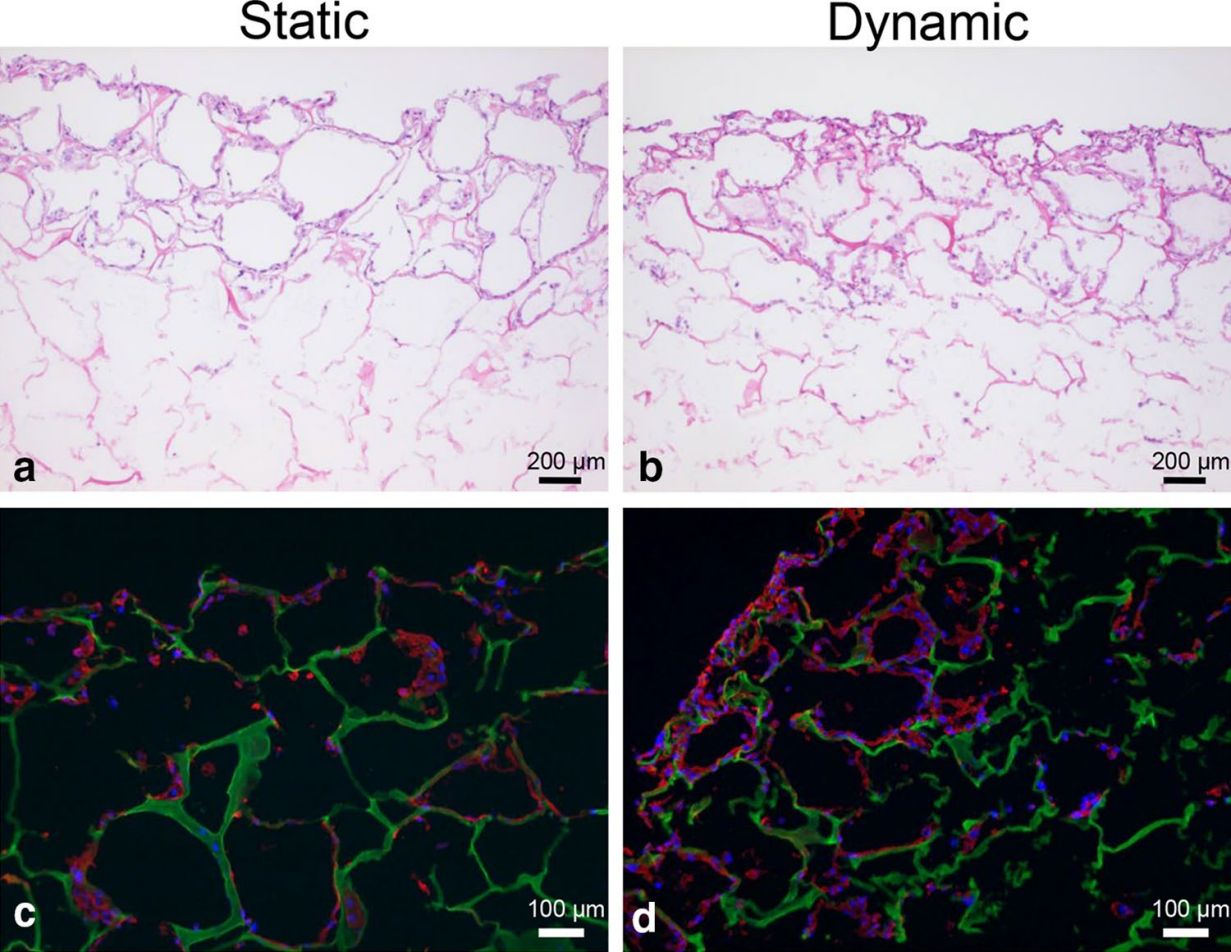
Representative Hematoxylin & Eosin (H&E) staining and immunofluorescence staining of the urothelial cell (UC) seeded constructs cultured under static (**a** and **c**) and dynamic (**b** and **d**) conditions. **a** and **b** H&E staining and c, d cytokeratin staining. *Green* collagen, *blue* nuclear stain (DAPI), *red* cytokeratin

UC lined the honeycomb and lamellae structures of the scaffold more prominent when cultured under static conditions compared to the UC cultured under dynamic stress (Fig. 6). Phenotypic analysis revealed strong expression of RCK103 regardless of the culture conditions (Fig. 6c, d). As expected, urothelial cells did not express any of the determined markers (data not shown). WST-1 showed no differences in UC proliferation between static and dynamic culture. Finally, SEM analysis showed more ruptures in the confluent UC layer which was exposed to uniaxial strain, compared to the static cultures (Fig. 5c, d).

## Discussion

One of the biggest challenges in tissue engineering is to create (preconditioned) templates that adequately mimic the native tissue at the phenotypic and organizational level. For bladder tissue engineering, cell seeded bladder dome-shaped scaffolds have been used in patients with variable results [13]. Until now, no method has proven to generate superior functional scaffolds for bladder tissues. Mechanical stimulation during scaffold preparation may improve the functional outcome. It has been used to condition engineered tissue based on the assumption that mimicking the physiological conditions of the native tissue improves its function once implanted to correct or replace lost tissue. In this study we show that mechanical stimulation of a porcine cell seeded collagen scaffold using cyclic uniaxial strain, mimicking the filling and emptying of the bladder results in increased smooth muscle cell growth, improved cell distribution, and more importantly SMC alignment, mimicking SMC organization in muscle fibers. Southgate et al. [14] showed that pig and human bladders have anatomical and biological similarities, making porcine derived bladder cells an adequate model for developing tissue engineering strategies.

Collagen-based scaffolds have been used extensively for bladder tissue engineering in (pre)clinical studies: immune responses are lacking and this natural biomaterial is highly cyto- and biocompatible. Nevertheless, despite these favourable characteristics implementation of collagen-based scaffolds is hampered by the poor and slow ingrowth of SMC after implantation [15, 16]. Whereas collagen scaffolds are replaced by newly formed tissue in 3–4 weeks, SMC ingrowth is limited to the rim of the defect. Enhanced interconnectivity of collagen scaffolds by decreasing the collagen density can lead to an improved SMC distribution [17]. Here we show that another possibility to improve SMC distribution is scaffold conditioning through pre-seeding and dynamic culturing of the construct. The current stimulation protocol was based on a pilot experiment where we compared slow, fast and “bladder like” stimulation protocols. The cellular distribution and cell density were superior. Most likely, the rapid stretch and or release induced too rapid material deformations, leading to cell detachment. Since we are using this technique to prepare in vitro conditioned scaffolds to function in the bladder, it may be that a “bladder like” protocol would be more effective. A possible refinement to prevent cell loss could involve a distinct filling (stretch) and extended emptying (release) phase.

Conditioning of the seeded construct by uniaxial stimulation resulted in SMC alignment. Moreover, SMC growing under mechanical stimulation showed higher α-SMA, desmin and calponin1 expression compared to static cultures as judged by PCR. Moreover, α-SMA, desmin and calponin-1 levels were higher by immunohistochemistry. This is in agreement with Ahvaz et al. [18] who also observed increased α-SMA and calponin-1 expression in SMC which were subjected to continuous stretch–relaxation cycles. Remarkably, the same investigators reported decreased α-SMA and calponin-1 expression using a different (synthetic) substrate [10]. In experiments lasting 6 h, calponin-1 and α-SMA were down regulated during smooth muscle cell stimulation [19]. These discrepancies may be related to differences in cell source, passage number, medium type, mechanotransduction frequencies or physical and chemical properties of the scaffolds.

It has been hypothesized that mechanical stimuli also trigger surface stretch receptors and adhesion sites of cells, resulting in the activation of genes which are responsible for the synthesis and secretion of extracellular matrix components, such as collagens [20]. Since we used bovine derived collagen as a template for our cells, we were unable to investigate the production of type I collagen. When we analyzed type III collagen production, we did not observe any influence of culture conditions on type III collagen expression. This contrasts with the observation that in static cultures and non-compliant bladders the type III collagen production is decreased [21, 22]. It is possible that longer culture times are required to observe increased collagen production. Other investigators have demonstrated that after longer culture periods (up to 14 days) type I and III collagen production levels increased in dynamic cultures [23].

The urothelium has an important osmotic barrier function: it protects the submucosa from environmental harm, mainly the toxic components of the urine. In our studies minor morphological and proliferation differences were observed between static and dynamic cultures, albeit that small cracks were present in the dynamic cultured urothelium. The small distortions that we observed in the UC layer are most likely due to the strain exerted on the collagen. Apparently SMC can accommodate this strain better than the UC. The absence of an adjacent lamina propria and/or fibroblasts in our in vitro model may indeed explain our observations. In the urinary bladder the UC are anchored on this superficial layer which contains both type IV collagen and elastin. Construction of collagen scaffolds in which UC and SMC are intimately connected will require a longer culture period. Alternatively, myofibroblasts could be used or dynamic culturing of SMC followed by a static culture of UC.

Augmentation cystoplasty with gastrointestinal tissue is a relatively safe and effective way to restore bladder capacity. However, this treatment can be associated with several complications including infections, stone formation, metabolic abnormalities and carcinogenesis. Tissue engineering may provide an alternative approach, but current methods have shown substantial side effects and suboptimal results. This may be related to the lack of mechanical stimulation during preparation, which can lead to fibrosis and poor compliance [13, 24]. The bladder is continuously filled and emptied, resulting in continuous loading of the tissue. By mimicking these dynamics during preparation of tissue engineered constructs a better outcome may be achieved. In addition, scaffold survival is heavily dependent on the formation of a new vascular bed. This is especially important when considering that large constructs are needed for relevant augmentation. To solve this, multiple smaller scaffolds instead of one large scaffold may be used (Roelofs et al., submitted). Thus, implantation of multiple conditioned scaffolds may be an attractive alternative for bladder augmentation using gastro-intestinal tissue; an assumption that needs to be tested in patients.

A potential drawback of the current study is that we only examined the effects of linear stretching on the cells while bladder filling causes a multi-directional strain. Whether multidirectional conditioning leads to a different outcome is unclear and bioreactor experiments along these lines may be of use. Moreover, a more physiological filling and contraction profile may be needed to create an optimally conditioned template. Finally, healthy bladder cells may not be available, and a different cell source may be required. Recently, stem cells derived from urine or adipose tissue have been successfully differentiated to functional smooth muscle cells for bladder tissue engineering applications. In addition, these stem cells can be harvested from the patient relatively easy, providing an autologous cell source [25, 26].

In summary, our results show that conditioning of collagen-based scaffolds by mechanical stimulation leads to more SMC with a more differentiated phenotype which may bypass difficulties related to poor SMC in-growth and muscle development in tissue engineered bladders. This brings us closer to our goal to engineer flexible tissues such as urinary bladder as an alternative to current graft tissues.

## Electronic supplementary material

Below is the link to the electronic supplementary material.

**Online Resource 1** Representative immunofluorescence staining and Scanning Electron Microscopy (SEM) of isolated porcine urothelial cells (UC) and bladder smooth muscle cells (SMC). UC stained with RCK103 (a), and αSMA expressing SMC (c) demonstrating homogenous cell populations. Scanning electron microscopic pictures of the used UC (b) and SMC (d) (250x) (TIF 18045 KB)
