# Supplementary figures and images for: The effect of a cyclic uniaxial strain on urinary bladder cells
Source: World J Urol. 2017 Feb 23;35(10):1531–9. doi: 10.1007/s00345-017-2013-9 (PMC5613063; doi:10.1007/s00345-017-2013-9)

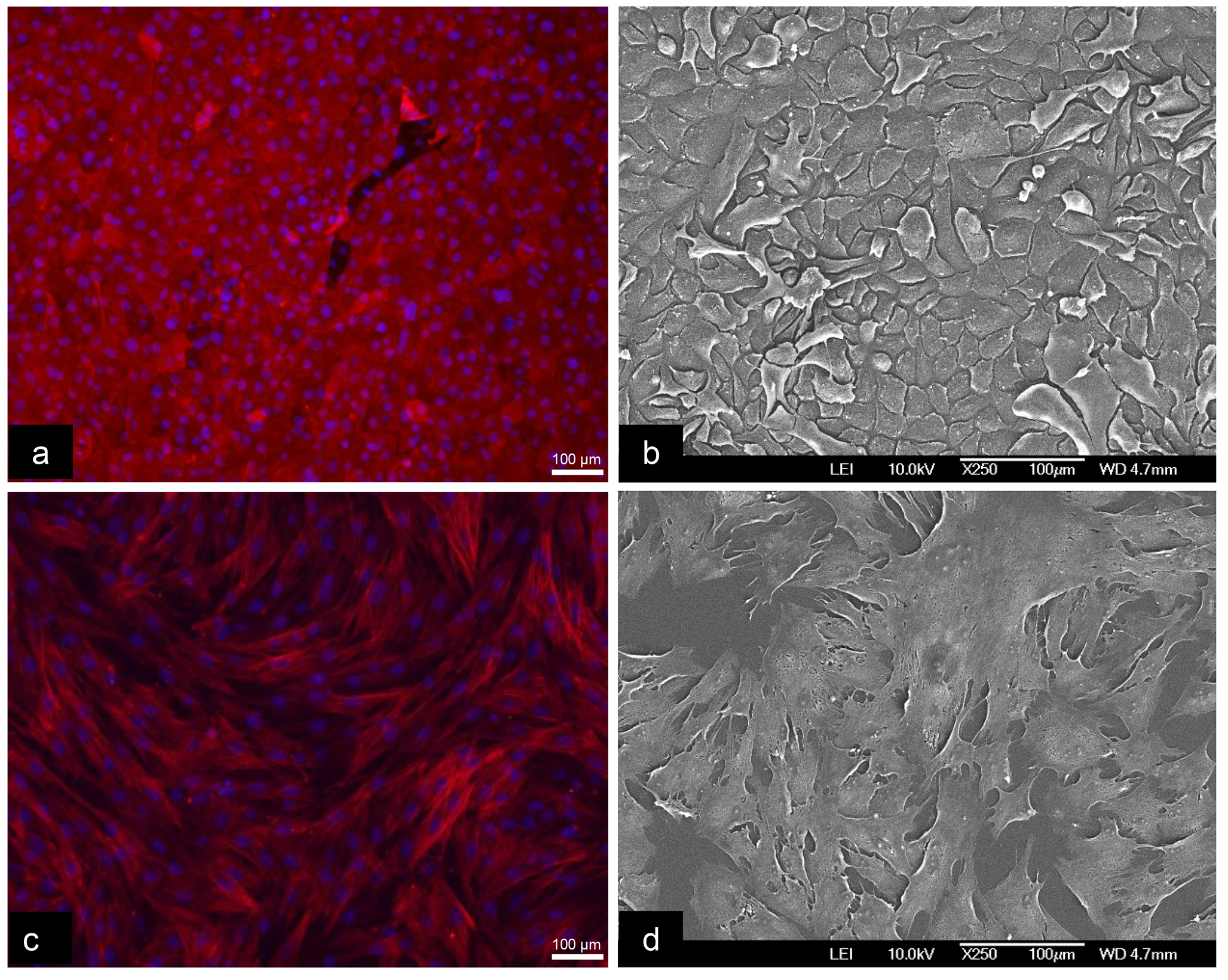

Supplement: Supplementary file 1 — Online Resource 1 Representative immunofluorescence staining and Scanning Electron Microscopy (SEM) of isolated porcine urothelial cells (UC) and bladder smooth muscle cells (SMC). UC stained with RCK103 (a), and αSMA expressing SMC (c) demonstrating homogenous cell populations. Scanning electron microscopic pictures of the used UC (b) and SMC (d) (250x) (TIF 18045 KB) [file 345_2017_2013_MOESM1_ESM.tif]
